# Targeted-Lymphoma Drug Delivery System Based on the Sgc8-c Aptamer

**DOI:** 10.3390/cancers15030922

**Published:** 2023-02-01

**Authors:** Estefanía Sicco, Hugo Cerecetto, Victoria Calzada, María Moreno

**Affiliations:** 1 Área de Radiofarmacia, Centro de Investigaciones Nucleares, Facultad de Ciencias, Universidad de la República, Montevideo 11400, Uruguay; 2 Departamento de Desarrollo Biotecnológico, Instituto de Higiene, Facultad de Medicina, Universidad de la República, Montevideo 11600, Uruguay

**Keywords:** lymphoma, aptamer, drug delivery, aptamer-drug conjugates, biotherapeutics, PTK7

## Abstract

**Simple Summary:**

Aptamers are oligonucleotides that recognise their target with high specificity and affinity, having properties comparable to those of antibodies; however, they present important advantages in terms of their size, production, and modification. These characteristics make them excellent candidates for the development of new biotechnological platforms and their application as imaging or therapy agents. The Sgc8-c aptamer binds to PTK7, allowing the recognition of haemato-oncological malignancies, among others. Thus, we have developed aptamer-drug conjugates by chemical synthesis, hybridizing Sgc8-c and dasatinib, a drug proposed for lymphoma chemotherapy. Here, we demonstrated that the aptamer-drug conjugate, **Sgc8-c-carb-da**, specifically inhibited lymphocyte growth, produced cell death, caused cell proliferation arrest, and affected mitochondrial potential. In addition, **Sgc8-c-carb-da** showed higher (2.5-fold) cytotoxic effects than dasatinib in an in vitro cell-directed assay that mimics in vivo conditions. These findings provide proof-of-concept of the therapeutic value of **Sgc8-c-carb-da** for lymphoma, creating new opportunities for the chemical synthesis of novel targeted biotherapeutics.

**Abstract:**

Aptamers are emerging as a promising new class of functional nucleic acids because they can specifically bind to any target with high affinity and be easily modified chemically with different pharmacophoric subunits for therapy. The truncated aptamer, Sgc8-c, binds to tyrosine-protein kinase-like 7 receptor, a promising cancer therapeutic target, allowing the recognition of haemato-oncological malignancies, among others. We have previously developed aptamer-drug conjugates by chemical synthesis, hybridizing Sgc8-c and dasatinib, a drug proposed for lymphoma chemotherapy. One of the best-characterised Sgc8-c-dasatinib hybrids, namely **Sgc8-c-carb-da**, was capable of releasing dasatinib at an endosomal-pH. Herein, we probed the therapeutic potential of this aptamer-drug conjugate. **Sgc8-c-carb-da** specifically inhibited murine A20 B lymphocyte growth and produced cell death, mainly by late apoptosis and necrosis. In addition, **Sgc8-c-carb-da** generated an arrest in cell proliferation, with a cell cycle arrest in the Sub-G1-peak. The mitochondrial potential was altered accordingly to these pathways. Moreover, using an in vitro cell-targeting assay that mimics in vivo conditions, we showed that **Sgc8-c-carb-da** displayed higher (2.5-fold) cytotoxic effects than dasatinib. These findings provide proof-of-concept of the therapeutic value of **Sgc8-c-carb-da** for lymphoma, creating new opportunities for the chemical synthesis of targeted biotherapeutics.

## 1. Introduction

Aptamers are oligonucleotides that recognise their target with high affinity and specificity and have properties comparable to antibodies; however, they have significant advantages in terms of size, production, and chemical modifications [1,2,3]. Therefore, they are excellent candidates for the development of new biotechnological platforms for applications as imaging or therapeutic agents [3,4,5,6]. 

Sgc8-c is a DNA aptamer, a truncated form of Sgc8, which has 41 bases and specifically binds to the tyrosine-protein kinase-like 7 (PTK7) receptor [7], also known as colon carcinoma kinase 4 (CCK4). This receptor, which acts as a co-receptor in several cell pathways, is a tumour biomarker that is overexpressed in different types of leukaemia, gastric tumours, colon, lung, breast, and prostate cancers, and even in metastases [8,9,10,11,12]. Likewise, it is involved in the migration and endothelial invasion of tumour cells [8,13,14].

Our research group has studied the truncated DNA aptamer Sgc8-c as a tool for the development of tumour imaging agents [15,16,17,18,19,20,21]. We found that Sgc8-c was able to recognise the PTK7 receptor in vivo in murine melanoma and lymphoma models. These findings provided us with the basis to develop potential therapeutic agents, using this truncated aptamer to direct anti-tumour drugs specifically to the site of action via the PTK7 receptor. 

The use of aptamers as probes for the selective delivery of drugs for clinical use has already been described in the literature [22,23]. Among the first examples, the use of DNA-intercalating drugs, like doxorubicin and daunorubicin, has been reported [22,24,25]; however, due to the intercalation process, such agents could affect aptamer target recognition, leading to a loss of specificity. A series of covalent binding strategies between the drug and the aptamer at a non-relevant site for recognition have been explored to avoid bioactivity loss [26]. Partially stable covalent bonds were used, such as amides [26]. Nevertheless, these constructions do not ensure the release of the drug under physiological conditions, potentially losing the activity provided by the drug component of the molecule. We recently described a molecular hybridisation strategy to covalently incorporate the anti-tumour agent dasatinib into the structure of Sgc8-c to generate potential biotherapeutics for haemato-oncology [27]. 

**Figure 1:**
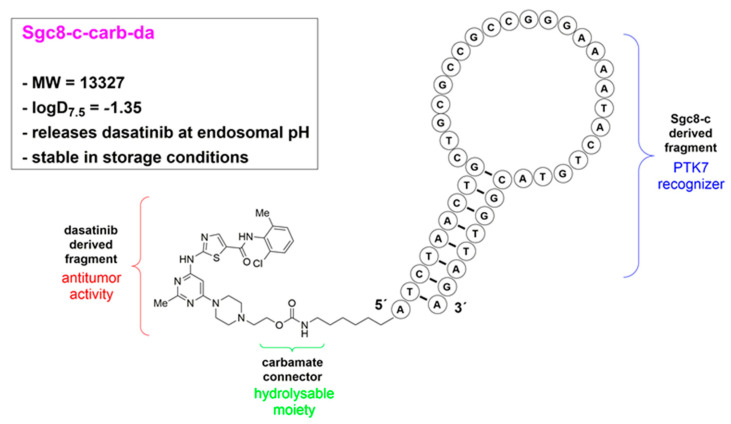
Schematic representation of **Sgc8-c-carb-da** structure and characteristics. Fragments correspond to anti-tumour activity (dasatinib-derived moiety), target recognition (Sgc8-c-derived fragment), and carbamate connectors (hydrolysable at endosomal pH [27]).

Dasatinib is a BCR-ABL kinase inhibitor approved by the Food and Drug Administration (FDA; USA) for the treatment of chronic myelogenous leukaemia and Philadelphia chromosome-positive acute lymphoblastic leukaemia [28]. We analysed different covalent connectors between dasatinib and the truncated aptamer and found that a carbamate moiety was able to preferentially release the drug at endosomal pH, which is optimal as the endosome is the Sgc8-c cellular uptake route [27]. Thus, we envisioned the generated hybrid agent, **Sgc8-c-carb-da** (Figure 1), to be highly attractive for therapeutic intervention. Therefore, we selected a lymphoma model to evaluate the potential of **Sgc8-c-carb-da** as a proof-of-concept for an aptamer-drug conjugate (ApDC) for biotherapy.

## 2. Material and Methods

### 2.1. Chemical Components

Dasatinib was purchased from Hong Kong Guokang Bio-Technology Co., Ltd. (Baoji City, China). 

The 5′-(6-aminohexyl)-modified Sgc8-c aptamer [12813 Da, 5′-(aminohexyl) ATC TAA CTG CTG CGC CGC CGG GAA AAT ACT GTA CGG TTA GA -3′, Sgc8-c-NH_2_] was purchased from IDT Technologies (Coralville, IA, USA).

**Sgc8-c-carb-da** (Figure 1) was synthesised and purified as described previously [27]. Briefly, dasatinib was coupled to a reactive pool, carbonate [29]. This intermediate product, dasatinib phenyl carbonate, was dissolved in dimethyl sulfoxide (DMSO). The aptamer Sgc8-c-NH_2_ was dissolved in a sodium phosphate buffer (0.1 M) and a sodium bicarbonate buffer (0.1 M) (50:50 *v*:*v*) (pH 8.3) and then mixed with dasatinib phenyl carbonate, and the mixture was maintained at 60 °C. After 1 h, N,N-dimethylformamide was added, and the reaction was maintained at 60 °C for 47 h. The reaction was stopped by washing **Sgc8-c-carb-da** with Milli-Q water using Microcon^®^ centrifugal filters. Finally, the product was purified by reversed-phase high-performance liquid chromatography. 

Water was purified and deionised (18 MΩ/cm^2^) in a Milli-Q water filtration system (Millipore Corp., Milford, UK).

### 2.2. Biological Components

All cell lines were obtained from the American Type Culture Collection (ATCC; Manassas, VA, USA): *Mus musculus* B lymphoma A20 cell line (TIB-208), *Homo sapiens* acute lymphoblastic leukaemia CCRF-CEM cell line (CCL-119) as the PTK7-positive control [30], and *Homo sapiens* glioblastoma U87 MG cell line (HTB-14) as the PTK7-negative control [31]. A20 and CCRF-CEM were cultured in Roswell Park Memorial Institute (RPMI)-1640 medium (Sigma-Aldrich, St. Louis, MO, USA) supplemented with 10% (*v*/*v*) foetal calf serum (FCS; Capricorn, Ebsdorfergrund, Germany) and 2 mM L-glutamine (Sigma-Aldrich) at 37 °C with 5% CO_2_. U87 MG was grown in Dulbecco’s Modified Eagle’s Medium (DMEM; Capricorn) supplemented with 10% FCS and 2 mM L-glutamine.

### 2.3. In Vitro Biological Assays

#### 2.3.1. Cytotoxicity Assay

Cells (5 × 10^4^ cells/well) were seeded in a 96-well plate and cultured for 48 h at 37 °C in 5% CO_2_, with different concentrations of **Sgc8-c-carb-da**, Sgc8-c, or dasatinib, ranging from 400 to 80,000 nM. In addition, cells were incubated with 20% DMSO (Sigma-Aldrich) and culture medium as controls. Then, the medium was removed, and cells were washed with 1X phosphate buffered saline (PBS), and 5 mg/mL of 3-(4,5-dimethylthiazol-2-yl)-2,5-diphenyltetrazolium bromide (MTT; Sigma-Aldrich) was added to each well. The plates were incubated for 4 h at 37 °C in 5% CO_2_. To dissolve MTT crystals, a 20% sodium dodecyl sulfate (SDS) solution (Sigma-Aldrich) was added to the plates and further incubated overnight at room temperature in dark conditions. Finally, the plates were read at 570 nm to determine the optical density (OD) in each well. Cell cytotoxicity (%) was calculated using the following formula: [(OD in the studied condition—OD with DMSO)/(OD in control medium—OD with DMSO)] × 100. The half maximal inhibitory concentrations (IC_50_), defined as the concentrations that induce 50% cytotoxicity, were determined from the viability vs. concentration curves using the software GraphPad8 (version 8.0.1).

#### 2.3.2. Washing Method

To study the role of Sgc8-c as a delivery system, we used an adaptation of a previously described Washing Method (WM) [32], which mimics the in vivo conditions. Cells were incubated with the studied agents (**Sgc8-c-carb-da**, Sgc8-c, or dasatinib) for 30, 60, or 120 min to allow specific binding to PTK-7. Afterward, the media was removed, and the cells were washed with PBS. Then, cells were incubated at 37 °C in the corresponding culture medium for up to 48 h. The MTT assay was developed as described above.

#### 2.3.3. Cell Death Studies

Induction of cell death was studied by flow cytometry to detect early and late apoptosis and necrosis using Annexin V conjugated to FITC (AV; BD Pharmingen) and propidium iodide (PI; Sigma-Aldrich). For this, cells (5 × 10^5^ cells/well in 24-well plates) were incubated with 200, 400, and 800 nM of **Sgc8-c-carb-da** or dasatinib for 24 h at 37 °C with 5% CO_2_. The washing method was also carried out in these studies to evaluate the role of Sgc8-c as a delivery system.

After the incubation period, the cells and supernatant were washed with cold FACS buffer (1× PBS, 2% FCS, and 25 mM EDTA), then centrifuged at 1800 rpm for 3 min at 4 °C. After that, the pellets were resuspended in 200 μL of FACS buffer and incubated with 2 μL of AV for 30 min in ice. Two more washes were performed with cold FACS buffer, and cells were further incubated with 5 μL of IP for 5 min. Samples were acquired in a FACS Canto II (BD Biosciences, San Diego, CA, USA). The results were analysed using FACS Diva (version 6.1.3) and FlowJo software (version 7.6).

#### 2.3.4. Mitochondrial Membrane Potential Assay

Changes in the mitochondrial membrane potential (Δψm) were analysed using the cationic carbocyanine dye JC-1. A20 cells were seeded in a 24-well plate (5 × 10^5^ cells/well) and incubated with 200, 400, and 800 nM of **Sgc8-c-carb-da** or dasatinib for 24 h at 37 °C with 5% CO_2_. To assess the potential of Sgc8-c as a delivery system, after 1 h of incubation with the compounds, cell cultures were washed with PBS and further incubated in the culture medium for 24 h. Likewise, cells were incubated with 1000 nM of the uncoupler carbonyl cyanide-*p*-trifluoromethoxy phenylhydrazone (FCCP; Sigma-Aldrich) for 15 min at 37 °C, as a positive control (100% JC-1 monomer retention, background of J-aggregate formation), or with culture medium, as a negative control (100% J-aggregate formation, background of JC-1 monomers). All samples were washed with PBS, centrifuged at 1800 rpm for 3 min, and incubated with 500 nM of 5,5′,6,6′-tetrachloro-1,1′,3,3′-tetraethylbenzimidazolylcarbocyanine iodide (JC-1; Sigma-Aldrich, St. Louis, MO, USA) [33,34] in culture medium for 30 min at 37 °C. Cells were washed again with PBS and analysed by flow cytometry, determining green monomers (Ex 485 nm/Em 535 nm) and red J-aggregates (Ex 560 nm/Em 595 nm). Ten thousand events were acquired and analysed using the Diva and FlowJo software (version 7.6). The percentage of JC-1 monomer retention was calculated using the following formula: % JC-1 monomer in the studied condition—% JC-1 monomer FCCP. Likewise, the percentage of J-aggregate formation was calculated using the following formula: % J-aggregates in the studied condition—% J-aggregates in control media.

#### 2.3.5. Arrest of Cell Proliferation Assay

To evaluate cell proliferation, cells (2.6 × 10^5^ cells/mL) were stained with 3 µM of the fluorescent compound succinimidyl-carboxyfluorescein ester (CFSE; Sigma-Aldrich) for 10 min at 37 °C with 5% CO_2_. Afterward, cells were washed with 1 mL of FCS for every 3 mL of cell suspension, then with PBS. Cells were resuspended in the corresponding medium, seeded in 24-well plates, and incubated with the IC_50_ of the agents (**Sgc8-c-carb-da** or dasatinib, determined for each of the cell lines) and two serial dilutions. In addition, cells were incubated with 20% DMSO or culture medium as controls for cell arrest and proliferation, respectively. Once again, the washing method was performed to assess the potential of Sgc8-c as a delivery system. After 48 h, cells and supernatants were collected together, washed with PBS, and analysed by flow cytometry. For each sample, 10,000 events were acquired in a FACS Canto II. The results were analysed using FACS Diva and FlowJo software. Cell proliferation (%) was calculated using the following formula: [(% cell proliferation in the studied condition—% cell proliferation with DMSO)/(% cell proliferation in control medium—% cell proliferation with DMSO)] × 100. 

#### 2.3.6. Cell Cycle Analysis by DNA Content

A20 cells were seeded in 24-well plates (5 × 10^5^ cells/well) and incubated with 200, 400, and 800 nM of **Sgc8-c-carb-da** or dasatinib at 37 °C with 5% CO_2_ for 24 h. To determine the role of Sgc8-c as a delivery system, samples were washed with PBS after one hour of incubation to remove compounds and further incubated with only the culture medium until 24 h were completed. Then, all samples were fixed with cold 70% ethanol at 4 °C for 2 h. To avoid the formation of lumps and guarantee cell fixation, the cell suspension was added dropwise to the ethanol with continuous, gentle shaking. Samples were washed twice with PBS and centrifuged at 2000 rpm at 4 °C for 3 min, then incubated with 0.1 mg/mL of Ribonuclease A (RNAse A; from bovine pancreas, Sigma-Aldrich, St. Louis, MO, USA) in 0.1% Triton X-100 (in PBS) at 37 °C for 30 min. After that, samples were stained with 5 µL of PI in cold RNAse buffer and incubated at 4 °C for 5 min. Samples were analysed by flow cytometry, acquiring 10,000 events, and the height, width, and area of the PI curve were acquired to perform an adequate analysis [35] using FACS Diva (version 6.1.3) and FlowJo software (version 7.6).

### 2.4. Statistical Analysis

Statistical analysis was performed using the Student’s *t*-test, and the *p* values of significance are indicated in each figure.

## 3. Results and Discussion

### 3.1. **Sgc8-c-carb-da** Displays Cytotoxic Activity against A20 Cells

**Figure 2:**
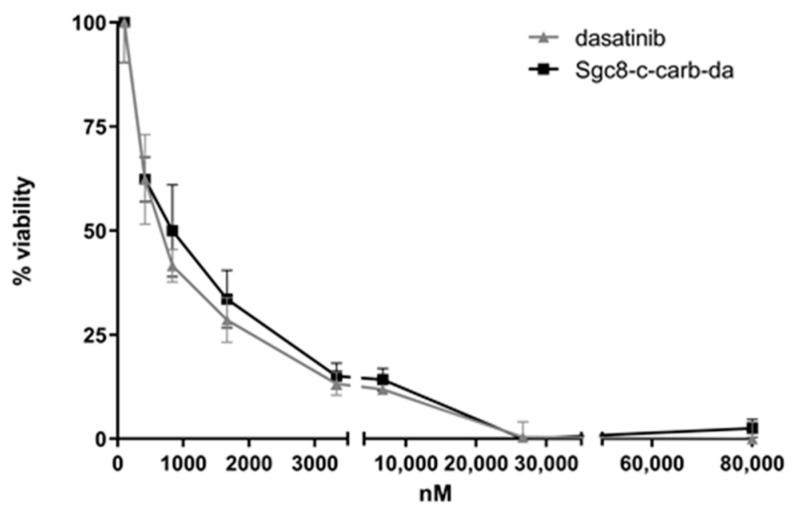
Doses-response curves for dasatinib and **Sgc8-c-carb-da** in A20 cells using the MTT method. Cytotoxicity was evaluated by MTT. Graph shows mean ± standard deviation (SD), n = 10 per condition.

The capability of **Sgc8-c-carb-da** to act as a biotherapeutic agent was determined. First, its cytotoxic potential was evaluated against A20 lymphoma cells by MTT assay, showing an IC_50_ of 820 nM (Figure 2, Table 1). In parallel, the cytotoxic potential of dasatinib against A20 cells was assessed (IC_50_ of 740 nM) (Figure 2, Table 1), demonstrating that **Sgc8-c-carb-da** maintained the cytotoxic activity of dasatinib (IC_50_,_dasatinib_/IC_50_,**_Sgc8-c-carb-da_** = 0.91) (Figure 2, Table 1). Therefore, this drug-delivery system would not affect the biological activity of dasatinib. Additionally, dasatinib does not have the ability to intercalate with DNA since it is not a pi-rich system [36,37]. Therefore, we expect no topological modifications of the aptamer and, consequently, no loss of target recognition. 

To further test whether **Sgc8-c-carb-da** activity was the result of each component of the hybrid agent (i.e., dasatinib and Sgc8-c), the cytotoxicity of Sgc8-c was also evaluated, being higher than 80,000 nM (Table 1, Figure S1A in Supplementary Materials). These results clearly showed that the **Sgc8-c-carb-da** cytotoxic activity against A20 lymphocytes is the result of the dasatinib component since the truncated aptamer Sgc8-c did not display cytotoxic activity in the experimental assayed conditions. Hence, Sgc8-c was not included in the following mechanistic experiments.

As expected, the IC_50_ for **Sgc8-c-carb-da** in PTK7-negative U87 MG cells [31] was higher than in A20 cells (IC_50_ 16,350 nM) (Table 1, Figure S1C in Supplementary Materials), and no difference was observed for **Sgc8-c-carb-da** and dasatinib activities (IC_50,dasatinib_/IC_50,**Sgc8-c-carb-da**_ = 1.23), with both IC_50_ values being extremely high. These cells are considered negative controls, as they are not susceptible to any of these agents.

On the other hand, the IC_50_ for **Sgc8-c-carb-da** in PTK7-positive CCRF-CEM cells [30] was also higher than in A20 cells (IC_50_ 7477 nM) (Table 1, Figure S1B in Supplementary Materials), revealing that this biotherapeutic agent is less effective in these cells, albeit CCRF-CEM cells express the PTK7 receptor, unlike U87 MG cells. 

However, **Sgc8-c-carb-da** displayed almost 2-fold higher activity than dasatinib (IC_50,dasatinib_/IC_50,**Sgc8-c-carb-da**_ = 1.64), suggesting the relevance of the PTK7 receptor in facilitating drug delivery into these cells, which are less sensitive to dasatinib. 

**Figure 3:**
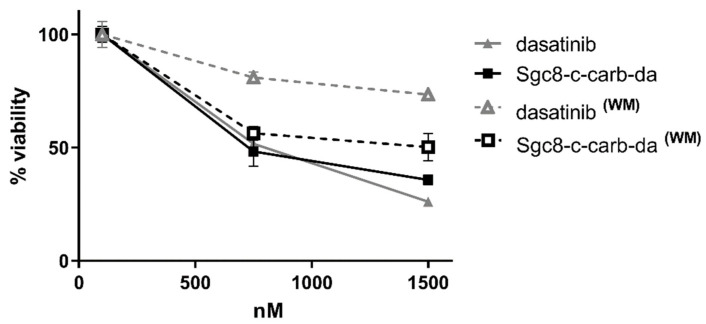
Evaluation of **Sgc8-c-carb-da** binding via PTK7 in A20 cells. Dose-response curves for dasatinib or **Sgc8-c-carb-da** after 48 h of incubation with A20 cells, with (WM) or without the washing method (60 min of drug exposure followed by a wash and further incubation). Cytotoxicity was evaluated by MTT. Graph shows mean ± SD, n = 10 per condition.

The lack of differences in cytotoxic activity between dasatinib and the new ApDC (IC_50_,_dasatinib_/IC_50_,**_Sgc8-c-carb-da_** = 0.91) could suggest the absence of Sgc8-c’s functional effect. In order to investigate whether the cellular binding of **Sgc8-c-carb-da** via PTK7 enhances its bio-response, an in vitro cell-binding study was performed. For that, a washing protocol was applied (see Materials and Methods Section 2.3.2), where cells were incubated with different concentrations of **Sgc8-c-carb-da** for 30, 60, or 120 min to allow binding to PTK7, then the assay media containing the biotherapeutic agents were removed, and the cells were washed. The IC_50_ values were determined as described before [32] (Table 2, Figure S2 in Supplementary Materials). **Sgc8-c-carb-da** increased the activity of dasatinib against A20 cells in all studied incubation times (Table 2, Figure 3, and Figure S2A,D in Supplementary Materials), being four times more cytotoxic after 120 min of incubation. Even at shorter incubation periods, the cytotoxic activities expressed as IC_50,dasatinib_/IC_50,**Sgc8-c-carb-da**_, were higher (2.82 and 2.42 for 30 and 60 min, respectively). Moreover, **Sgc8-c-carb-da** showed an increase in cytotoxic activity in PTK7-positive CCRF-CEM cells compared to dasatinib, particularly after 120 min of incubation (IC_50,dasatinib_/IC_50,**Sgc8-c-carb-da**_ = 2.08, Table 2, Figure S2B,E in Supplementary Materials). However, **Sgc8-c-carb-da** did not display different activity than dasatinib, with or without the washing step, in PTK7-negative U87 MG cells (IC_50,dasatinib_/IC_50,**Sgc8-c-carb-da**_ ~ 1, Table 2, Figure S2C,F in Supplementary Materials). The reduced IC_50,**Sgc8-c-carb-da**_ in these cells could be explained by the lack of receptor mediation in the biopharmaceutical uptake (IC_50_,**_Sgc8-c-carb-da_** 16,350 nM versus 32,580 nM after 48 h and 120 min of incubation, respectively), confirming that this limited activity is PTK7-independent.

Altogether, these results show the potential of **Sgc8-c-carb-da** as a biotherapeutic agent since it promotes A20 cell cytotoxicity in a PTK-7-mediated manner; however, the MTT assay is a comprehensive measurement of cell cytotoxicity and reflects many altered pathways, including cell death, cell proliferation arrest, and changes in mitochondria dynamics, among others. Therefore, the next experiments were addressed to unravel the mechanisms behind **Sgc8-c-carb-da**’s cytotoxic effect.

### 3.2. **Sgc8-c-carb-da** Promotes A20-Cell Apoptosis and Necrosis

**Figure 4:**
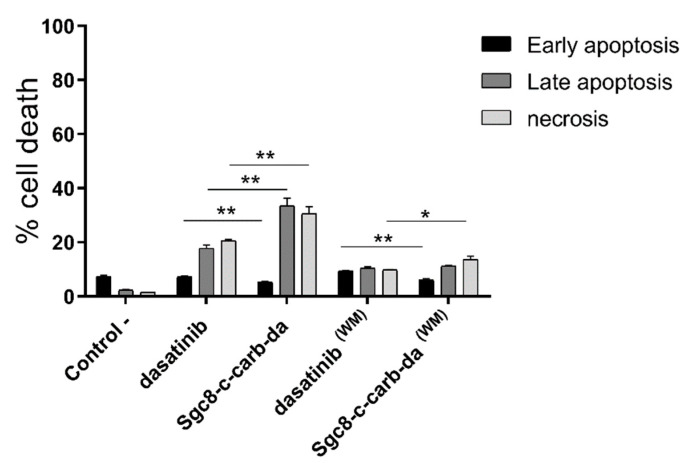
Percentage of cell death of A20 cells obtained after incubation with 800 nM dasatinib or **Sgc8-c-cab-da** for 24 h, with (WM) or without the washing method (60 min of drug exposure followed by a wash and further incubation). Graph shows mean ± SD, n = 3 per condition. * *p* < 0.05, ** *p* < 0.01 (Student’s *t*-test).

The mechanisms of A20 cell death (i.e., early and late apoptosis and necrosis) promoted by **Sgc8-c-carb-da** were assessed by flow cytometry using AV and PI. For that, A20 cells were incubated with 800 nM of **Sgc8-c-carb-da** or dasatinib in the same conditions as described above. After 24 h of incubation, 45.6% of dasatinib-treated A20 cells died, mainly by late apoptosis (17.8%) and necrosis (20.6%), whereas 69.3% of **Sgc8-c-carb-da**-treated cells died, triggering a significant increase in late apoptosis (33.6%, *p* < 0.01) and necrosis (30.5%, *p* < 0.01) (Figure 4). Note 11.2% of cell death was observed in the control conditions. When A20 cells were exposed to the biomolecules for only 60 min, then washed away and incubated for another 23 h, a reduction in cell death was observed (*p* < 0.01). Dasatinib induced early apoptosis (9.5%) as much as late apoptosis (10.4%) and necrosis (9.9%), while for **Sgc8-c-carb-da,** early apoptosis was significantly lower (6.0%, *p* < 0.01), slightly shifting the death process toward late apoptosis (11.2%) and especially to necrosis (13.6%, *p* < 0.05). 

**Figure 5:**
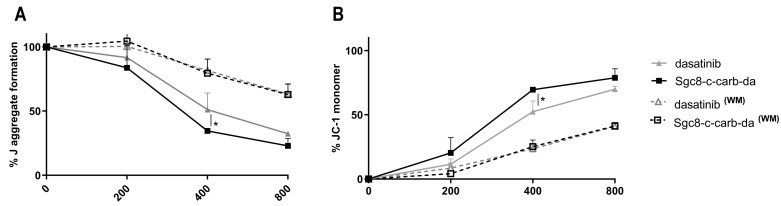
Changes in mitochondrial membrane potential induced by **Sgc8-c-carb-da**. Quantification of the JC-1 assay obtained from incubation of the A20 cell line with 200, 400, and 800 nM of dasatinib or **Sgc8-c-carb-da** for 24 h, with (WM) or with the washing method (60 min of drug exposure followed by a wash and further incubation). (**A**) Percentages of J-aggregate formation and (**B**) percentages of JC-1 monomer retention are shown. Graph shows mean ± SD, n = 3 per condition. * *p* < 0.05 (Student’s *t*-test).

To assess whether the observed cell death was preceded by alterations in mitochondrial structure and transmembrane potential, changes in mitochondrial membrane potential were analysed using the JC-1 assay. In living cells (normal ΔΨM), the JC-1 dye enters and accumulates in the mitochondria and spontaneously forms red fluorescent J-aggregates. Conversely, in unhealthy (apoptotic) cells (altered ΔΨM), the JC-1 dye does not reach sufficient concentration at the mitochondrial to promote J-aggregate formation, thus retaining its original green fluorescence [38]. This assay was performed after the treatment of A20 cells with different doses of **Sgc8-c-carb-da** or dasatinib, as described above. **Sgc8-c-carb-da** caused a decrease in J-aggregate formation (Figure 5A) and an increase in the retention of JC-1 monomers, slightly greater than that of dasatinib in a dose-dependent manner (Figure 5B); however, no differences were observed when cells were treated with **Sgc8-c-carb-da** or dasatinib for only 60 min, washed, and later incubated for another 23 h (Figure 5A,B). These results showed a Δψm that is indicative of a mitochondrial membrane compromise, in agreement with previous data for dasatinib in other cellular systems [39]. This behaviour could be related to the activation of cellular pathways, particularly apoptosis, which is associated with the response to this drug and generates an uncoupling of the mitochondrial potential [33,34]. In this regard, no differences in the percentage of (early plus late) apoptotic cells were observed when cells were treated with **Sgc8-c-carb-da** or dasatinib for only 60 min, washed, and later incubated for another 23 h (Figure 4).

### 3.3. **Sgc8-c-carb-da** Triggers Cell Proliferation Arrest, Mainly in the subG1 Phase

**Figure 6:**
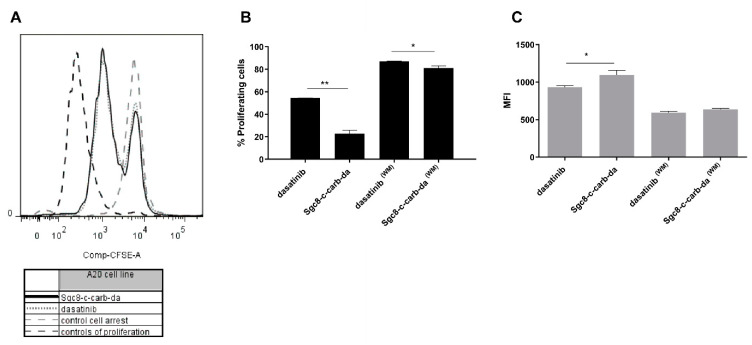
Arrest of cell proliferation in A20 cells after incubation with **Sgc8-c-carb-da** and dasatinib (800 nM) for 48 h, with (WM) or without the washing method (60 min of drug exposure followed by a wash and further incubation). (**A**) Cell proliferation histogram, controls of proliferation, and cell arrest: incubation with medium (100% proliferating cells) and 20% of DMSO (0% proliferating cells), respectively. (**B**) Percentages of proliferating cells are shown. (**C**) Mean fluorescence indices (MFI) for CFSE. Graph shows mean ± SD, n = 3 per condition. * *p* < 0.05, ** *p* < 0.01 (Student’s *t*-test).

Along with cell death, we assessed the potential of **Sgc8-c-carb-da** to promote cell proliferation arrest. A20, CCRF-CEM, and U87 MG cells were stained with CFSE to track cell division. In each cell division, daughter cells will receive half of the CFSE label of their parent cell and so on, allowing the tracking of different cell generations. Thus, when cells proliferate, CFSE fluorescence decreases. CFSE-stained tumour cells were treated with the IC_50_ of **Sgc8-c-carb-da** or dasatinib for 48 h. The results showed that both compounds caused the arrest of A20- and CCRF-CEM-cell proliferation, revealed by a higher percentage of cells that did not undergo division (Figure 6A and Figure S3A in Supplementary Materials). On the other hand, no differences were observed between U87 MG cells treated with **Sgc8-c-carb-da** or dasatinib with the control condition (Figure S3B in Supplementary Materials).

The percentage of proliferating cells was lower, and concordantly, the CFSE mean fluorescence index (MFI) was higher after treatment with 800 nM **Sgc8-c-carb-da** than dasatinib for 48 h (Figure 6B,C). Likewise, when A20 cells were incubated for 60 min (washing method), **Sgc8-c-carb-da** promoted a higher cell arrest than dasatinib, with the percentage of proliferative cells being 81.0% and 87.0% for **Sgc8-c-carb-da** and dasatinib, respectively (Figure 6B). These observations could be explained by the ability of the aptamer to interact with PTK7 and facilitate the delivery of dasatinib intracellularly, arresting cell proliferation.

**Figure 7:**
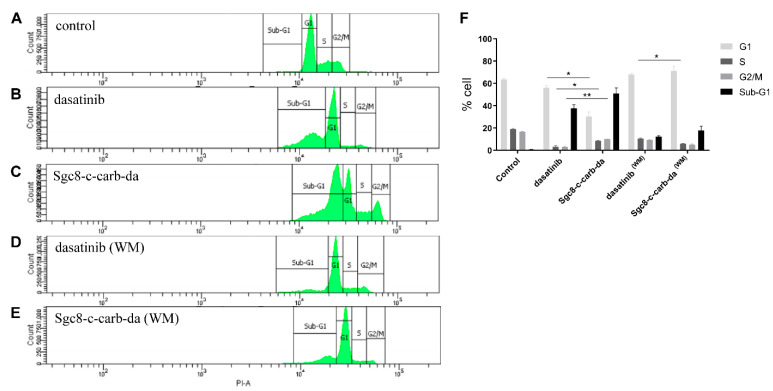
Analysis of cell cycle phases. Assay was performed on A20 cells after incubation with 800 nM **Sgc8-c-carb-da** and dasatinib for 24 h, with (WM) or without the washing method (60 min of drug exposure followed by a wash and further incubation). Histograms of cells incubated with (**A**) control culture medium, (**B**) dasatinib for 24 h, (**C**) **Sgc8-c-carb-da** for 24 h, (**D**) dasatinib for 60 min then washed, and (**E**) **Sgc8-c-carb-da** for 60 min then washed. (**F**) Percentages of cells in the different cell cycle phases after incubation under the mentioned conditions. Graph shows mean ± SD, n = 3 per condition. * *p* < 0.05, ** *p* < 0.01 (Student’s *t*-test).

Since A20 cell proliferation arrest was detected upon **Sgc8-c-carb-da** treatment, the cell cycle distribution was assessed via flow cytometry. **Sgc8-c-carb-da** generated significant changes in the cell cycle with respect to dasatinib, observing a decrease in the G1, S, and G2/M phases and an increase in the Sub-G1 (Figure 7A–C,F). After 24 h of incubation with **Sgc8-c-carb-da** (800 nM), 51.10% of the cell population had stopped in Sub-G1, while 30.30% of the cells were in G1, 8.60% in S, and 10.15% in the G2/M phases (Figure 7C,F). This cell cycle distribution differed from that found after 24 h of incubation with dasatinib (800 nM) (37.60% of the cell population stopped in Sub-G1, while 56.05% were in G1, 3.25% in S, and 3.00% in the G2/M phases; Figure 7B,F). The Sub-G1 peak is associated with cell apoptosis [40], confirming that **Sgc8-c-carb-da** facilitated dasatinib-mediated apoptosis. In line with previous assays, when A20 cells were incubated with **Sgc8-c-carb-da** (800 nM) for only 60 min, followed by a washing method, only 17.85% of the cell population was arrested in Sub-G1, while 71.20% of the cells were in G1, 5.75% in S, and 5.15% in the G2/M phases (Figure 7D–F). These analyses confirmed that both agents have similar effects on the cell cycle progression in a dose-dependent manner (Figure S4 in Supplementary Materials).

## 4. Conclusions

Here, we report the potential **Sgc8-c-carb-da** ApDC, which combines a truncated aptamer that recognises the PTK7 receptor as a target-specific component and dasatinib sub-structure as a cytotoxic component. The site-specific drug attachment was achieved by covalent conjugation via a carbamate moiety of the amino aptamer to the dasatinib primary alcohol [27]. One of the relevant properties of **Sgc8-c-carb-da** is its capability to release dasatinib in a pH-dependent manner, with the endosome (pH 5.0) being the optimal place for drug release. This property is highly relevant because it implies some advantages to traditional treatment. On the one hand, Sgc8-c aptamer-based probes have significant tumour uptake and blood clearance [16,19,21]. Hence, the in vivo use of **Sgc8-c-carb-da** would facilitate drug delivery to the site of action with minimal hydrolysis at the non-target sites, enhancing drug availability at the tumour site and reducing systemic toxicity. On the other hand, since the endosomal route has been confirmed for some aptamers’ internalisation [16,41,42], and particularly endosomal Sgc8-c-uptake in A20 cells [15], once **Sgc8-c-carb-da** penetrates lymphoma cells, dasatinib release would take place, facilitating drug delivery at the appropriate site of action. Thus, this system merits further exploration in haemato-oncology indications.

The objective of the current study was to establish the potential of **Sgc8-c-carb-da** as an ApDC on A20 lymphoma cells. Here, we demonstrated these hypotheses: (i) the dasatinib does not undergo biological activity loss; (ii) higher cytotoxic activity is observed in the PTK7-over-expressing cells (A20 and CCRF-CEM vs. U87 MG cells); and (iii) the ApDC displays higher activity than dasatinib in a washing method set-up, highlighting the relevance of Sgc8-c as a delivery system.

In addition, we explored the mode of action of this ApDC in different biological-behaviour studies (i.e., the PTK7-dependence of cytotoxic effects, cell death pathways, and cell cycle perturbation) and compared it to the drug alone. **Sgc8-c-carb-da** reveals significant cytotoxicity against A20 cells, potentiating dasatinib biological activity. **Sgc8-c-carb-da** promotes cell death by necrosis and apoptosis, accompanied by changes in the mitochondrial membrane potential. It also generates cell proliferation arrest, which results in an increase in the Sub-G1 peak and decreases in the S and G2/M phases. Collectively, these results confirm the hypothesis that the Sgc8-c aptamer acts as a tumour-specific vehicle for dasatinib, binding to PTK7 and delivering the drug into the cell, enhancing its biological activity. To further translate the Sgc8-c platform as a drug delivery system into tumours, there is still a need for pre-clinical and, ultimately, clinical investigations.

## Figures and Tables

**Table 1:** Cytotoxic effects of **Sgc8-c-carb-da**, dasatinib, and Sgc8-c against A20 cells, PTK7-positive CCRF-CEM, and negative U87 MG controls, expressed as IC_50_.

	IC_50_ (nM)			IC_50,dasatinib_/IC_50,Sg8-c-carb-da_
Cell Line	Sgc8-c-carb-da	Sgc8-c	Dasatinib	
A20	820 ± 30	>80,000	740 ± 20	0.91
CCRF-CEM	7477 ± 80	>80,000	12,260 ± 30	1.64
U87 MG	16,350 ± 30	>80,000	20,160 ± 70	1.23

**Table 2:** Cytotoxic effects of dasatinib and **Sgc8-c-carb-da** against A20 cells, PTK7-positive CCRF-CEM, and negative U87 MG controls after different drug exposure times, expressed as IC_50_.

Exposure Time (min)	IC_50_ (nM)		IC_50,dasatinib_/IC_50,Sgc8-c-carb-da_
A20	Dasatinib	Sgc8-c-carb-da	
30	1070 ± 20	380 ± 20	2.82
60	1650 ± 80	680 ± 130	2.43
120	1750 ± 20	440 ± 20	3.98
CCRF-CEM			
30	18,460 ± 40	15,110 ± 20	1.22
60	11,800 ± 20	9520 ± 20	1.24
120	12,650 ± 20	6060 ± 20	2.08
U87 MG			
30	30,120 ± 20	31,310 ± 40	0.96
60	33,950 ± 30	32,230 ± 10	1.05
120	32,010 ± 20	32,580 ± 20	0.98

## Data Availability

The data presented in this study are available in this article.

## Supplementary Materials

The following supporting information can be downloaded at: https://www.mdpi.com/article/10.3390/cancers15030922/s1, Figure S1: Dose-response curves for dasatinib, **Sgc8-c-carb-da**, and Sgc8-c-NH_2_ using the MTT method in (A) A20, (B) CCRF-CEM, and (C) U87 MG cells. Figure S2: Dose-response curves for dasatinib or **Sgc8-c-carb-da** after 48 h of incubation, with (WM) or without the washing method (60 or 120 min of drug exposure followed by a wash and further incubation), in the different cell lines: (A) A20 (60 min), (B) CCRF-CEM (60 min), (C) U87 MG (60 min), (D) A20 (120 min), (E) CCRF-CEM (120 min) and (F) U87 MG (120 min). Cytotoxicity was evaluated by MTT. Graph shows mean ± SD, n = 10 per condition. Figure S3: Arrest of cell proliferation induced by **Sgc8-c-carb-da** and dasatinib in (A) CCRF-CEM and (B) U87 MG cell lines. Figure S4: Phases of the cell cycle induced by **Sgc8-c-carb-da** and dasatinib in A20 cells. Assay performed on the A20 cells after incubation with 200, 400, and 800 nM **Sgc8-c-carb-da** and dasatinib for 24 h, with (WM) or without the washing method (60 min of drug exposure followed by a wash and further incubation). Graph shows mean ± SD, n = 3 per condition.

## References

[1] Qu N., Ying Y., Qin J., Chen A.K. (2021). Rational design of self-assembled RNA nanostructures for HIV-1 virus assembly blockade. Nucleic. Acids Res..

[2] Yamada K., Hildebrand S., Davis S.M., Miller R., Conroy F., Sapp E., Caiazzi J., Alterman J.F., Roux L., Echeverria D. (2021). Structurally constrained phosphonate internucleotide linkage impacts oligonucleotide-enzyme interaction, and modulates siRNA activity and allele specificity. Nucleic. Acids Res..

[3] Qi S., Duan N., Khan I.M., Dong X., Zhang Y., Wu S., Wang Z. (2022). Strategies to manipulate the performance of aptamers in SELEX, post-SELEX and microenvironment. Biotechnol. Adv..

[4] Calzada V. (2019). Aptamers in Diagnostic and Molecular Imaging Applications. Aptamers in Biotechnology.

[5] Tong R., Coyle V.J., Tang L., Barger A.M., Fan T.M., Cheng J. (2010). Polylactide nanoparticles containing stably incorporated cyanine dyes for in vitro and in vivo imaging applications. Microsc. Res. Tech..

[6] Liu M., Wang L., Lo Y., Shiu S.C., Kinghorn A.B., Tanner J.A. (2022). Aptamer-Enabled Nanomaterials for Therapeutics, Drug Targeting and Imaging. Cells.

[7] Shangguan D., Tang Z., Mallikaratchy P., Xiao Z., Tan W. (2007). Optimization and Modifications of Aptamers Selected from Live Cancer Cell Lines. ChemBioChem.

[8] Ganier L., Morelli X., Borg J.-P. (2020). Rôle en cancérologie et ciblage du récepteur à activité tyrosine kinase PTK7. Méd./Sci..

[9] Gärtner S., Gunesch A., Knyazeva T., Wolf P., Högel B., Eiermann W., Ullrich A., Knyazev P., Ataseven B. (2014). PTK 7 Is a Transforming Gene and Prognostic Marker for Breast Cancer and Nodal Metastasis Involvement. PLoS ONE.

[10] Zhang H., Wang A., Qi S., Cheng S., Yao B., Xu Y. (2014). Protein Tyrosine Kinase 7 (PTK7) as a Predictor of Lymph Node Metastases and a Novel Prognostic Biomarker in Patients with Prostate Cancer. Int. J. Mol. Sci..

[11] Lin Y., Zhang L.H., Wang X.H., Xing X.F., Cheng X.J., Dong B., Hu Y., Du H., Li Y.A., Zhu Y.B. (2012). PTK7 as a novel marker for favorable gastric cancer patient survival. J. Surg. Oncol..

[12] Golubkov V., Prigozhina N., Zhang Y., Stoletov K., Lewis J., Schwartz P., Hoffman R., Strongin A. (2014). Protein-tyrosine pseudokinase 7 (PTK7) directs cancer cell motility and metastasis. J. Biol. Chem..

[13] Berger H., Breuer M., Peradziryi H., Podleschny M., Jacob R., Borchers A. (2017). PTK7 localization and protein stability is affected by canonical Wnt ligands. J. Cell Sci..

[14] Shin W., Maeng Y., Jung J., Min J., Kwon Y., Lee S. (2008). Soluble PTK7 inhibits tube formation, migration, and invasion of endothelial cells and angiogenesis. Biochem. Biophys. Res. Commun..

[15] Castelli R., Ibarra M., Faccio R., Miraballes I., Fernández M., Moglioni A., Cabral P., Cerecetto H., Glisoni R.J., Calzada V. (2022). T908 polymeric micelles improved the uptake of Sgc8-c aptamer probe in tumor-bearing mice: A co-association study between the probe and preformed nanostructures. Pharmaceuticals.

[16] Sicco E., Mónaco A., Fernandez M., Moreno M., Cerecetto H. (2021). Metastatic and non-metastatic melanoma imaging using Sgc8-c aptamer PTK7-recognizer. Sci. Rep..

[17] Sicco E., Baez J., Ibarra M., Fernández M., Cabral P., Moreno M., Cerecetto H., Calzada V. (2020). Sgc8-c Aptamer as a Potential Theranostic Agent for Hemato-Oncological Malignancies. Cancer Biother. Radiopharm..

[18] Sicco E., Báez J., Margenat J., García F., Ibarra M., Cabral P., Moreno M., Cerecetto H., Calzada V. (2018). Derivatizations of Sgc8-c aptamer to prepare metallic radiopharmaceuticals as imaging diagnostic agents: Syntheses, isolations, and physicochemical characterizations. Chem. Biol. Drug Des..

[19] Calzada V., Moreno M., Newton J., González J., Fernández M., Gambini J.P., Ibarra M., Chabalgoity A., Deutscher S., Quinn T. (2017). Development of new PTK7-targeting aptamer-fluorescent and -radiolabelled probes for evaluation as molecular imaging agents: Lymphoma and melanoma in vivo proof of concept. Bioorganic. Med. Chem..

[20] Arevalo A.P., Castelli R., Ibarra M., Crispo M., Calzada V. (2022). In vivo evaluation of Sgc8-c aptamer as a molecular imaging probe for colon cancer in a mouse xenograft model. Int. J. Mol. Sci. Press.

[21] Calzada V., Báez J., Sicco E., Margenat J., Fernández M., Moreno M., Ibarra M., Gambini J., Cabral González P., Cerecetto H. (2017). Preliminary in vivo characterization of a theranostic aptamer: Sgc8-c-DOTA-67Ga. Aptamers.

[22] Yazdian-robati R., Arab A., Ramezani M., Abnous K., Mohammad S. (2017). Application of aptamers in treatment and diagnosis of leukemia. Int. J. Pharm..

[23] Patil S.D., Rhodes D.G., Burgess D.J. (2005). DNA-based therapeutics and DNA delivery systems: A comprehensive review. AAPS J..

[24] Bagalkot V., Farokhzad O.C., Langer R., Jon S. (2006). An Aptamer–Doxorubicin Physical Conjugate as a Novel Targeted Drug-Delivery Platform. Angew. Chemie. Int. Ed..

[25] Taghdisi S.M., Abnous K., Mosaffa F., Behravan J. (2010). Targeted delivery of daunorubicin to T-cell acute lymphoblastic leukemia by aptamer. J. Drug Target..

[26] Zhao N., Pei S.N., Qi J., Zeng Z., Iyer S.P., Lin P., Tung C.H., Zu Y. (2015). Oligonucleotide aptamer-drug conjugates for targeted therapy of acute myeloid leukemia. Biomaterials.

[27] Sicco E., Almeida L., Moreno M., Calzada V., Cerecetto H. (2021). Chemical conjugations of Sgc8-c with the lymphoma drug dasatinib to generate selective biotherapeutics. Aptamers.

[28] De Novellis D., Cacace F., Caprioli V., Wierda W.G., Mahadeo K.M., Tambaro F.P. (2021). The tki era in chronic leukemias. Pharmaceutics.

[29] Carpino L., Collins D., Göwecke S., Mayo J., Thatte S., Tibbetts F. (1973). t-Butyl carbazate. Organic Syntheses.

[30] Yin J., He X., Wang K., Xu F., Shangguan J., He D., Shi H. (2013). Label-Free and Turn-on Aptamer Strategy for Cancer Cells Detection Based on a DNA–Silver Nanocluster Fluorescence upon Recognition-Induced Hybridization. Anal. Chem..

[31] Jacobson O., Weiss I.D., Wang L., Wang Z., Yang X., Dewhurst A., Ma Y., Zhu G., Niu G., Kiesewetter D.O. (2015). 18F-Labeled Single-Stranded DNA Aptamer for PET Imaging of Protein Tyrosine Kinase-7 Expression. J. Nucl. Med..

[32] Alawak M., Abu Dayyih A., Mahmoud G., Tariq I., Duse L., Goergen N., Engelhardt K., Reddy Pinnapireddy S., Jedelská J., Awak M. (2021). ADAM 8 as a novel target for doxorubicin delivery to TNBC cells using magnetic thermosensitive liposomes. Eur. J. Pharm. Biopharm..

[33] Cossarizza A., Salvioli S. (2000). Flow Cytometric Analysis of Mitochondrial Membrane Potential Using JC-1. Curr. Protoc. Cytom..

[34] Perelman A., Wachtel C., Cohen M., Haupt S., Shapiro H., Tzur A. (2012). JC-1: Alternative excitation wavelengths facilitate mitochondrial membrane potential cytometry. Cell Death Dis..

[35] Hof J.V. (1973). Cell Cycle Analysis. Tissue Culture.

[36] Korashy H.M., Rahman A.F.M.M., Kassem M.G., Dasatinib (2014). Profiles of Drug Substances, Excipients and Related Methodology.

[37] Luo Y., Liao F., Lu W., Chang G., Sun X. (2011). Coordination polymer nanobelts for nucleic acid detection. Nanotechnology.

[38] Sivandzade F., Bhalerao A., Cucullo L. (2019). Analysis of the Mitochondrial Membrane Potential Using the Cationic JC-1 Dye as a Sensitive Fluorescent Probe. Bio-Protocol.

[39] Bouitbir J., Panajatovic M.V., Frechard T., Roos N.J., Krähenbühl S. (2020). Imatinib and Dasatinib Provoke Mitochondrial Dysfunction Leading to Oxidative Stress in C2C12 Myotubes and Human RD Cells. Front. Pharmacol..

[40] Yu R., Zhang Y., Xu Z., Wang J., Chen B., Jin H. (2018). Potential antitumor effects of panaxatriol against DU-15 human prostate cancer cells is mediated via mitochondrial mediated apoptosis, inhibition of cell migration and sub-G1 cell cycle arrest. J. BUON.

[41] Porciani D., Cardwell L.N., Tawiah K.D., Alam K.K., Lange M.J., Daniels M.A., Burke D.H. (2018). Modular cell-internalizing aptamer nanostructure enables targeted delivery of large functional RNAs in cancer cell lines. Nat. Commun..

[42] Xiao Z., Shangguan D., Cao Z., Fang X., Tan W. (2008). Cell-specific internalization study of an aptamer from whole cell selection. Chem.-A Eur. J..

