# Supplementary material for: Targeted-Lymphoma Drug Delivery System Based on the Sgc8-c Aptamer
Source: Cancers (Basel). 2023 Feb 1;15(3):922. doi: 10.3390/cancers15030922 (PMC9913644; doi:10.3390/cancers15030922)
Supplement: Supplementary file 1 [file cancers-15-00922-s001.zip › cancers-2098184-supplementary.pdf]

# Targeted-lymphoma drug delivery system based on the Sgc8-c aptamer.

Estefanía Sicco[1,2], Hugo Cerecetto[1], Victoria Calzada[1,*] and María Moreno[2,*]

## SUPPLEMENTARY MATERIALS

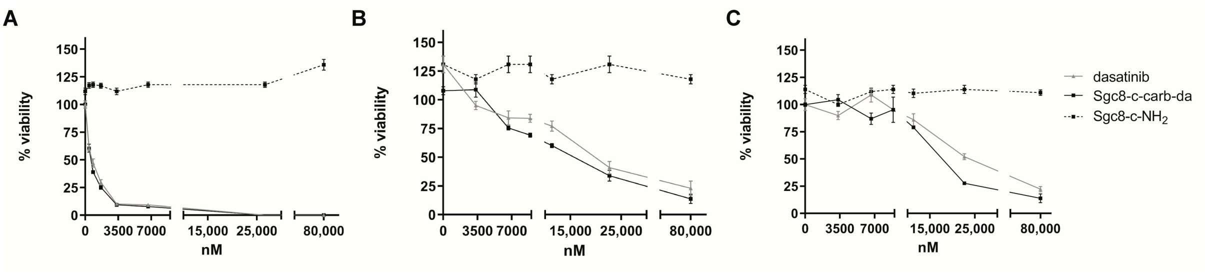


**Figure S1**: Dose-response curves for dasatinib, **Sgc8-c-carb-da,** and Sgc8-c-$NH_2$ using the MTT method in (**A**) A20, (**B**) CCRF-CEM, and (**C**) U87 MG cells. Graph shows mean ± SD, n = 10 per condition.

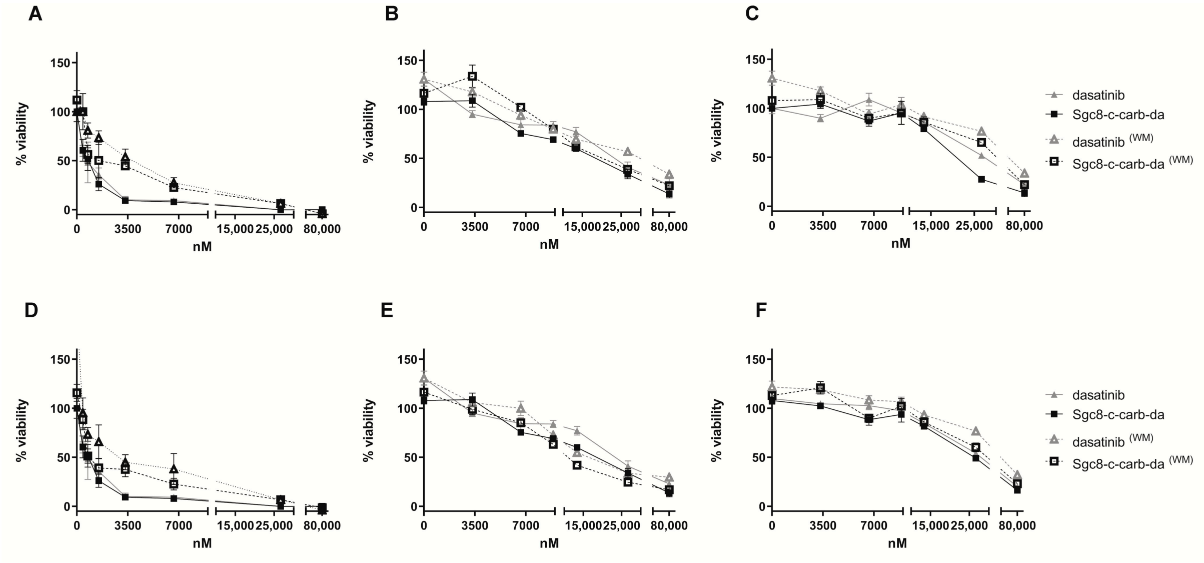


**Figure S2.** Dose-response curves for dasatinib or **Sgc8-c-carb-da** after 48 h of incubation, with (WM) or without the washing method (60 or 120 min of drug exposure followed by a wash and further incubation), with the different cell lines: (**A**) A20 (60 min), (**B**) CCRF-CEM (60 min), (**C**) U87 MG (60 min), (**D**) A20 (120 min), (**E**) CCRF-CEM (120 min), and (**F**) U87 MG (120 min). Cytotoxicity was evaluated by MTT. Graph shows mean ± SD, n = 10 per condition.

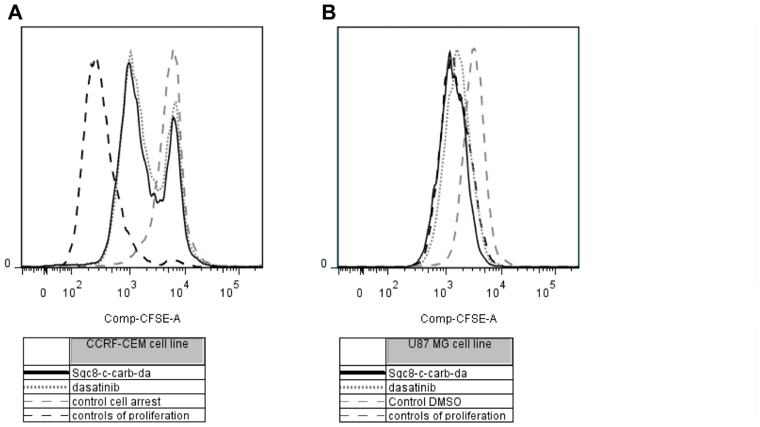


**Figure S3.** Arrest of cell proliferation induced by **Sgc8-c-carb-da** and dasatinib in (**A**) CCRF-CEM and (**B**) U87 MG cell lines. Cell proliferation arrest histogram, controls of proliferation, and cell arrest: incubation with medium (100% proliferating cells) and 20% of DMSO (0% proliferating cells), respectively.

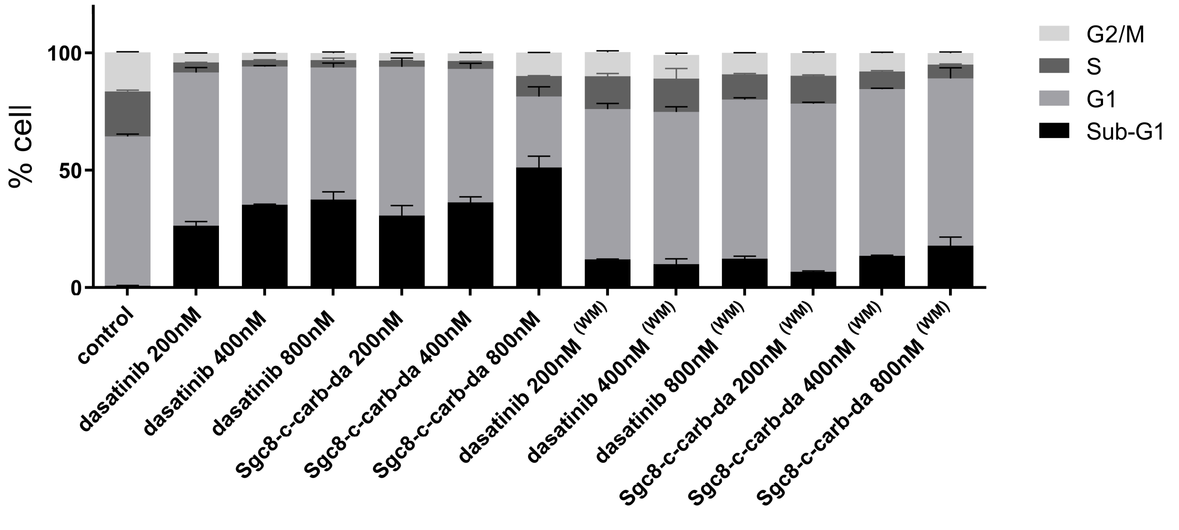


**Figure S4.** Phases of the cell cycle induced by **Sgc8-c-carb-da** and dasatinib in A20 cells. Assay performed on the A20 cells after incubation with 200, 400, and 800 nM **Sgc8-c-carb-da** and dasatinib for 24 h, with (WM) or without the washing method (60 min of drug exposure followed by a wash and further incubation). Graph shows mean ± SD, n = 3 per condition.
